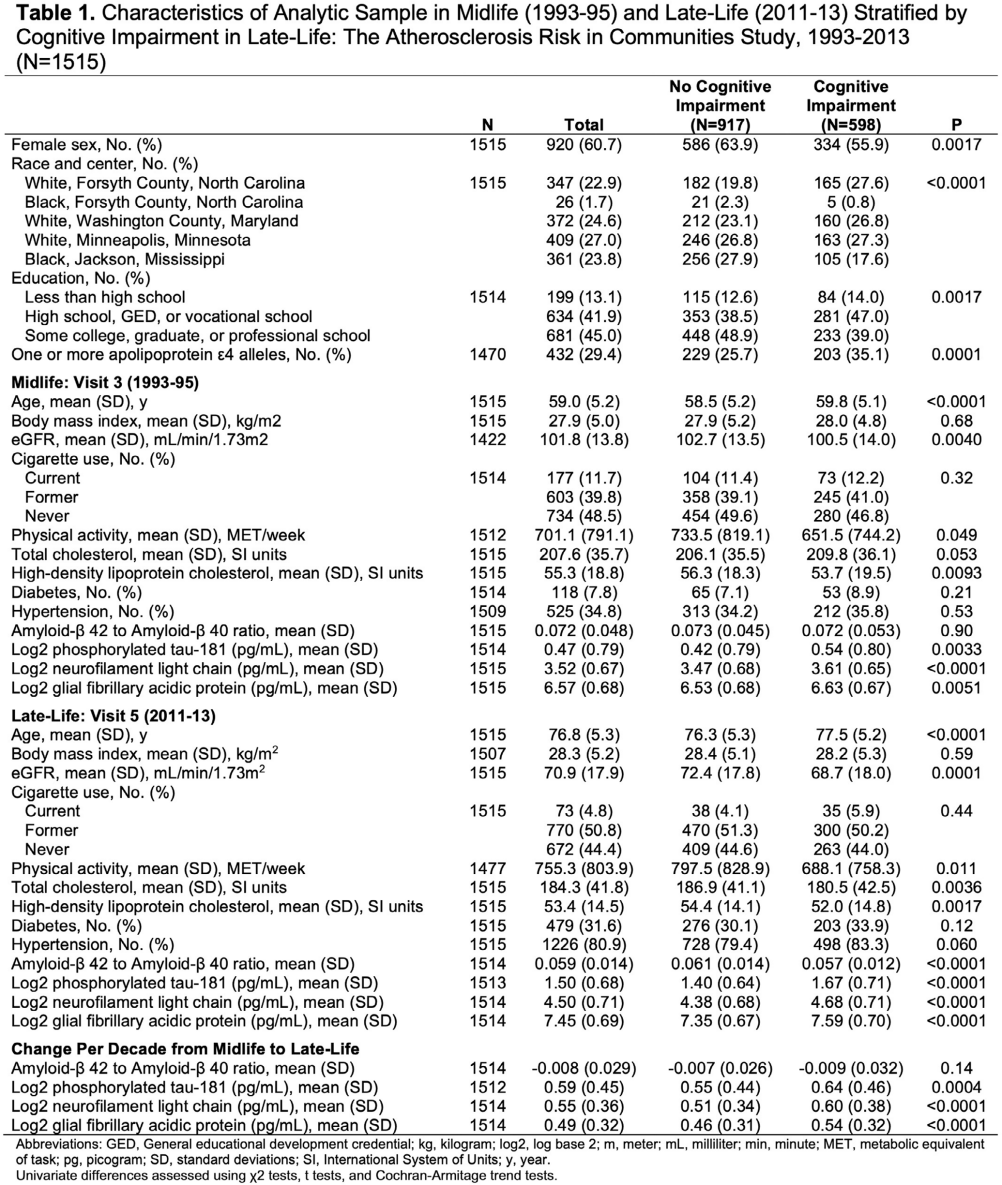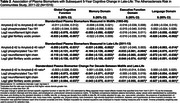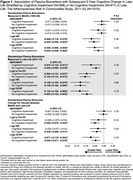# Midlife to Late‐Life Changes in Plasma Biomarkers of Alzheimer's Disease Pathology and Neurodegeneration and Associations with 5‐Year Cognitive Change in Late‐Life: The Atherosclerosis Risk in Communities (ARIC) Study

**DOI:** 10.1002/alz70856_106168

**Published:** 2026-01-08

**Authors:** Priya Palta, James Russell Pike, Yifei Lu, Keenan A. Walker, Kevin J. Sullivan, Gwen Gwen Windham, Bharat Thyagarajan, Michelle M Mielke, Pamela L. Lutsey, Timothy M. Hughes, David S. Knopman, A. Richey Sharrett, Rebecca F. Gottesman, Thomas H. Mosley, Josef Coresh

**Affiliations:** ^1^ University of North Carolina at Chapel Hill, Chapel Hill, NC, USA; ^2^ Departments of Population Health and Medicine, New York University Grossman School of Medicine, New York, NY, USA; ^3^ University of North Carolina Chapel Hill, Chapel Hill, NC, USA; ^4^ National Institute on Aging, National Institutes of Health, Baltimore, MD, USA; ^5^ University of Mississippi Medical Center, The MIND Center, Jackson, MS, USA; ^6^ University of Minnesota, Minneapolis, MN, USA; ^7^ Division of Public Health Sciences, Wake Forest University, School of Medicine, Winston‐Salem, NC, USA; ^8^ University of Minnesota School of Public Health, Minneapolis, MN, USA; ^9^ Wake Forest University School of Medicine, Winston‐Salem, NC, USA; ^10^ Mayo Clinic, Rochester, MN, USA; ^11^ Johns Hopkins University Bloomberg School of Public Health, Baltimore, MD, USA; ^12^ National Institute of Neurological Disorders & Stroke, Bethesda, MD, USA

## Abstract

**Background:**

Plasma biomarkers of Alzheimer's disease (AD) pathology and neurodegeneration provide a non‐invasive method to assess brain pathology associated with dementia and cognitive decline. However, it remains unclear whether these biomarkers are differentially associated with changes in specific cognitive domains or how they relate to subsequent cognitive change in participants with or without cognitive impairment.

**Methods:**

Plasma biomarkers were assayed in a subset of ARIC participants using Quanterix SiMoA technology on stored specimens collected in midlife (visit 3: 1993‐95, mean age: 59) and late‐life (visit 5: 2011‐13, mean age: 77). Biomarkers included amyloid‐β 42 to amyloid‐β 40 (Aβ42:Aβ40) ratio, phosphorylated tau at threonine 181 (*p*‐Tau181), neurofilament light (NfL), and glial fibrillary acidic protein (GFAP). Factor scores for global cognition and cognitive domains (memory, language, executive function) were calculated from ten neuropsychological tests administered at visits 5 (2011‐13), 6 (2016‐17), 7 (2018‐19), 8 (2020), and 9 (2021‐22). Linear mixed effects models with multiple imputation of pre‐death cognitive change estimated the association between biomarkers in midlife and late‐life, and biomarker change from midlife to late‐life, with 5‐year change in late‐life global and domain‐specific cognition, adjusting for demographics, lifestyle and cardiovascular risk factors, and APOE‐ε4 status. Exploratory analyses examined effect modification of the biomarker‐global cognition associations by visit 5 adjudicated cognitive impairment.

**Results:**

Among 1515 participants with plasma biomarkers (60.7% female, 25.5% identified as Black), 39% had late‐life cognitive impairment (Table 1). Higher midlife NfL and GFAP were associated with faster 5‐year declines in global cognition and language during late‐life (Table 2). Only midlife NfL was associated with faster late‐life memory decline. No midlife biomarkers were associated with executive function. In late‐life, lower Aβ42:Aβ40 and higher *p*‐Tau 181, NfL, and GFAP were associated with faster declines in global cognition, memory, and language. Midlife to late‐life changes in *p*‐Tau 181, NfL, and GFAP were associated with declines in global cognition and language. No consistent effect modification by cognitive impairment was observed (Figure 1).

**Conclusions:**

Plasma biomarkers of AD pathology, neuronal injury, and astrogliosis, measured at different life epochs (midlife vs. late‐life) show distinct association patterns with subsequent changes in cognitive function across domains.